# Acceptance and commitment therapy as a transdiagnostic approach to adolescents with different anxiety disorders: study protocol

**DOI:** 10.1007/s00787-024-02608-2

**Published:** 2024-11-14

**Authors:** Diana Vieira Figueiredo, Maria do Céu Salvador, Daniel Rijo, Paula Vagos

**Affiliations:** 1 Center for Research in Neuropsychology and Cognitive-Behavioral Intervention (CINEICC), Faculty of Psychology and Educational Sciences, University of Coimbra, Coimbra, Portugal; 2 Departamento de Educação E Psicologia, William James Research Center, Universidade de Aveiro, Aveiro, Portugal

**Keywords:** Acceptance and commitment therapy; Psychological flexibility/inflexibility; Adolescents; Anxiety disorders; Network analysis; Randomized controlled trial

## Abstract

Adolescence increases vulnerability to mental ill-health, particularly anxiety disorders like Social Anxiety Disorder (SAD) and Generalized Anxiety Disorder (GAD). Still, the understanding of Psychological Flexibility/Inflexibility (PF/PI) as a transdiagnostic framework relevant to explaining these difficulties and the efficacy of Acceptance and Commitment Therapy (ACT) in addressing adolescent SAD and GAD remains limited. Hence, this study has two aims: examining the role of PI/PF on adolescents’ mental health and assessing ACT’s efficacy for adolescent SAD and GAD. We will collect data from 400 community adolescents and employ network analysis to explore connections between PI/PF processes, anxiety, and flourishing. We will also investigate pathways linking PI/PF processes with flourishing and anxiety across samples (clinical SAD, clinical GAD, and mentally healthy groups), expecting to find similar patterns. Furthermore, a Randomized Controlled Trial with three groups (control, SAD experimental and GAD experimental), each comprising 29 participants, and four assessment moments (pre- and post-intervention and 3- and 6-months follow-up) will be conducted. Similar improvements are anticipated in both experimental groups, driven by changes in PI/PF processes. Findings from this work will provide valuable evidence to PI/PF as accurate conceptualizations of human suffering and expand the transdiagnostic application of ACT to adolescents with anxiety disorders.

**Supplementary Information:**

The online version contains supplementary material available at 10.1007/s00787-024-02608-2.

## Background

According to the World Health Organization [1], 14% of adolescents worldwide experience a mental health disorder, but lifetime prevalence estimates have reached as high as 49.5% [2]. Research consistently shows that anxiety disorders (ADs) are the most common mental health conditions during adolescence [2–8], with the age of onset usually occurring in childhood or adolescence [9–11]. The presence of ADs during those periods has been linked to an increased likelihood of experiencing anxiety during emerging adulthood [12], and to a greater risk of presenting other disorders (e.g., substance and alcohol abuse/dependence, or major depressive disorder,[13]. Adolescent ADs have also been linked to significant short and long-term impairments across a broad spectrum of life domains [13–16]. Experiencing ADs during adolescence can hinder the acquisition of crucial competencies to adolescents overall development that can shape their future lives and lead to negative outcomes in adulthood [17]. Hence, increasing the understanding of the common mechanisms underlying mental health problems, with a particular focus on different ADs in adolescence, and how these mechanisms can be used to sustain efficacious psychological interventions, is a crucial research concern.

Indeed, commonalities in psychological processes underlying human suffering and psychopathology have been increasingly considered, at both conceptual and therapeutic levels (e.g., [18, 19]), as alternatives to considering the distinct mechanisms associated with specific disorders. This shift in perspective has been driven by multiple factors, including empirical evidence from epidemiological studies pointing to high comorbidity rates among mental health diagnoses both in adult [10] and adolescent [8, 20] samples. Additionally, the tendency of mental health problems, including ADs, to predict and cascade both within and between disorders throughout individuals' lives has been consistently reported in the literature (e.g., [14, 21]). While disorder-specific interventions (i.e., interventions focused on a single diagnosis) seem to yield some unintended improvements in non-targeted comorbid disorders [22, 23], they may not comprehensively address the complex interplay and dynamics observed among mental health disorders. This overlap and continuity among and within classes of disorders indicate the presence of common underlying mechanisms, thereby suggesting that treatments should be conceived to address broader processes rather than focusing solely on the specificities of certain disorders.

Transdiagnostic approaches aim to identify common elements that cut across mental health disorders, thus better reflecting the complexity and dimensionality of the human experience, and more accurately representing the reality of mental health problems. These approaches may offer several advantages. For example, transdiagnostic interventions may be more efficacious in the treatment of comorbid disorders and in preventing future mental health conditions (e.g., [24, 25]). As such, a growing body of empirical research points to the efficacy of transdiagnostic interventions in multiple disorders (e.g. [25–30]) as well as their equivalence to diagnosis-specific interventions [31]. Moreover, transdiagnostic approaches do not rely on specific diagnosis categories outlined in the Diagnostic and Statistical Manual of Mental Disorders (5th ed., DSM-5; [32]), thus allowing to target symptoms associated with subclinical conditions. Transdiagnostic approaches may also have practical and economic benefits, including shorter treatment lengths and reduced need for future access to mental health services (e.g., [25, 33]). Furthermore, it may be easier to train clinicians in transdiagnostic approaches since a single protocol can be applied to multiple disorders, instead of requiring a separate protocol for each disorder [33].

Acceptance and Commitment Therapy (ACT) is a widely researched and empirically sustained transdiagnostic approach to behavior change (e.g., [18, 34–36]) addressing both psychopathology (suffering) and psychological health. Within this framework ([37]), psychological suffering (and mental health disorders) arises from the interdependent interactions between six core processes (Cognitive Fusion, Experiential Avoidance, Attachment to the Conceptualized Self, Dominance of a Conceptualized Past/Feared Future, Lack of Values Clarity, and Inaction, Impulsivity or Avoidant Persistence,see supplementary material for a detailed description of each process). Together, these processes sustain Psychological Inflexibility (PI), a rigid pattern in which internal experiences (e.g., thoughts, emotions) dominate over contextual cues, undermining the individuals’ ability to persist in and/or change behavior to pursue chosen values ([38]). Problematic behaviors associated with mental health problems may stem from the engagement in psychologically inflexible strategies (e.g., perceiving thoughts as literal representations of reality – i.e., cognitive fusion -, and/or avoiding specific relevant situations to minimize unwanted internal experiences—inaction, impulsivity or avoidant persistence driven by experiential avoidance) that reinforce one another, becoming more rigid and entrenched over-time, and ultimately narrowing the individuals’ lives. ACT aims to reverse PI processes through the cultivation of Psychological Flexibility (PF), which refers to the ability to be in contact with the present moment, regardless of unpleasant internal experiences, while adapting and persisting in behaviors aligned with valued life directions [39]. PF entails six interrelated processes, each representing the opposite of one of the PI processes: Cognitive Defusion, Acceptance, Self as Context, Contact with the Present Moment, Values, and Committed Action [38], see supplementary material for a detailed description of each process). Through the cultivation of PF, ACT does not aim to change the form or frequency of internal events. Rather, ACT intends to promote psychological health by changing how one relates to those events and reduce their behavioral impact, thus laying the foundation for actions that move individuals towards valued and meaningful living [38]. ACT’s perspective on psychological health is closely tied to the process of individual and idiosyncratic growth, which is defined by the gradually increasing choices individuals make to embrace the present and move towards a life worth living [39]. This perspective can be captured by the concept of flourishing, defined as a state of vitality associated with the ability to engage in functional behaviors, in meaningful relationships and to cope with the demands and stresses of life [40]. Flourishing may thus be a relevant construct indicative of desired behavior change towards valued life directions.

The parallel conceptualization of processes sustaining psychopathology (i.e., PI) and, alternatively, mental health and flourishing (i.e., PF) specified by the ACT model offers a functional dimensional approach to mental health problems. Moreover, it provides a model of treatment that enables the identification of different treatment components and of psychological mechanisms that may facilitate change [39]. Multiple meta-analyses with adult populations showed that ACT and ACT-based treatments were as effective as well-established evidence-based interventions (e.g., Cognitive Behavioral Therapy; CBT), and superior to placebo or waiting list control conditions and treatment as usual in treating depression, anxiety disorders, substance use disorders, somatic health problems and other forms of adult psychopathology (e.g., [35, 41, 42]). Regarding the mechanisms underlying change during ACT, a systematic review conducted by Stockton et al. [43] identified a lack of studies on this matter. The authors pointed that few studies examined the role of specific PF processes (e.g., committed action, values, and self-as-context) as potential mediators of change in ACT. Despite the limited research available, findings from this review suggested that PF, acceptance, and cognitive defusion mediated change in mental health outcomes while non-ACT specific processes (e.g., challenging dysfunctional cognitions) did not. Levin et al. [44] conducted a meta-analysis focused on laboratory-based component studies providing evidence for PF components impact on psychological outcomes. Specifically, significant positive effect sizes were observed for conditions promoting acceptance, defusion, present moment awareness, and values, compared to inactive conditions. Further sustaining the PI/PF model assumptions (i.e., the aim is not to directly change the form or frequency of internal events [45, 46]), larger effect sizes were observed for primary targeted outcomes (e.g., willingness to reengage in a difficult task) than for overall non-specific outcomes (e.g., intensity/frequency of negative thoughts and feelings). Network analysis studies also highlighted the theoretically proposed interconnectedness between PI/PF processes [47], and their association with mental health outcomes such as well-being/quality of life, internalizing symptoms [48, 49] and PTSD symptoms [50]. However, findings were inconsistent regarding the centrality of each PI/PF process in the network (e.g., [47, 49]). Considering the cumulative evidence, the ACT model seems to offer a suitable framework to conceptualize and intervene in adults’ suffering and mental health conditions.

Nevertheless, the role of PI/PF in adolescents’ mental health remains poorly understood, and little is known about the efficacy of ACT in this age group. Previous studies support the role of PI processes in adolescents’ suffering and psychopathology (e.g., [49–54]), as well as the role of PF in adolescents’ well-being [55, 56] and meaning in life [57]. These studies relied mostly on the Avoidance and Fusion Questionnaire—Youth Version (AFQ-Y), which is a widely used measure to assess PI and explore its relationship with psychopathology related variables (e.g., [58]). However, the AFQ-Y assesses PI uniquely in relation to cognitive fusion and experiential avoidance [59], and so the literature has been amiss in considering all six core processes of PF/PI. Failing to consider all these processes may result in a less specific and idiosyncratic perspective on the theoretical and applied suitability of PI/PF to adolescents' mental health.

At the therapeutic level, a meta-analysis by Fang & Ding [34] examined 14 studies with children and adolescents, and found support for the efficacy of ACT in multiple mental health problems. ACT outperformed waiting list control conditions and treatment as usual, and did not significantly differ from evidence based interventions (i.e., CBT) in improving negative outcomes such as anxiety and depression. Promising results have been found regarding ACT efficacy on adolescents mental health problems such as ADs [60], depressive symptoms [45, 46, 61, 62], obsessive–compulsive disorder [27–30, 63], social and school adaptation [64].

Despite the potential of ACT for promoting adolescents’ mental health, further research is needed to confidently establish its efficacy. Specifically, there is a scarcity of methodologically robust designs, including Randomized Controlled Trials (RCTs), investigating ACT interventions in this population (see [27–30, 65] for a review). Also, adolescence is a period marked by significant and rapid physical, psychological, and social changes [66], which underscores the importance of studies considering extended post-intervention follow-up periods to fully assess the utility of ACT for this age group. Moreover, because ACT's efficacy on different non-comorbid adolescent mental health problems is not clearly established, there is a gap in our understanding of ACT's transdiagnostic intervention principles (i.e., that targeting the same processes is helpful for different mental health difficulties). This gap hinders our ability to further address other complex issues in adolescent mental health, such as comorbidity and the cascading effects of mental health problems.

In light of this, it is important to understand how conceptualizations can better inform interventions to increase the well-being of adolescents with diverse ADs. Particularly, SAD and GAD are ADs that hold significant prevalence rates in adolescence: 12-month prevalence estimates of 4.8% [20] and point prevalence estimates of 9.4% [67] have been reported for SAD,12-month prevalence estimates of 2.1% [20] and 6-month prevalence estimates of 9.7% [3] have been reported for GAD. Both these disorders tend to reveal a chronic course that may fluctuate by evolving into other ADs or mood disorders in adulthood [21, 68, 69]. However, transdiagnostic approaches to these disorders in adolescence, particularly GAD, are still poorly investigated at both conceptual and therapeutic levels. ACT has been proven effective for SAD and GAD treatment in adults [68–73]. To our knowledge, only few studies pointed to ACT efficacy for SAD [60, 74, 75], and only one considered a sample that included adolescents with GAD [60]. Thus, research on the efficacy of ACT on adolescent SAD and GAD is still largely missing, and mechanisms underlying change have not yet been investigated.

### AIMS

Given the scarcity of research on the applicability of ACT’s conceptual premises to adolescents, the first aim of this study is to empirically test PI/PF as accurate conceptualizations of suffering and flourishing in adolescents. As stated in the report by the Association for Contextual Behavioral Science (ACBS) Task Force [76], understanding the relationships between processes as well as their specific associations with relevant outcomes may better guide the development and delivery of interventions based on PI/PF processes. To do so, we will:

- Explore PI/PF processes networks and their association with adolescents’ anxiety symptoms and flourishing in a community sample (Study I) to provide for an initial understanding of the structural relationships between PI/PF processes in adolescents and their experience of anxiety and flourishing. We expect PI/PF processes to present interconnected relationships between each other. Moreover, we expect all PF processes to positively associate with flourishing and negatively associate with anxiety, even when considering all processes simultaneously. Contrarily, all PI processes are expected to associate negatively with flourishing and positively with anxiety, also when considering all processes simultaneously. These expected results align with ACT’s conceptual model as well as with preliminary evidence found in adults (e.g., [47, 48]). Given the exploratory nature of this study, and the inconsistent and limited findings on the centrality of each PI/PF process (e.g., [47, 49]), no hypothesis were raised regarding the strongest and most influential ACT processes in the networks
- Investigate pathways linking PI/PF processes with flourishing and anxiety symptoms, and that models’ invariance across adolescents samples (clinical SAD, clinical GAD and mentally healthy groups; Study II). Given previous research on the associations between PI/PF and positive (e.g., well-being; e.g., [55, 57]) and negative (e.g., emotional dysregulation [52, 54],) mental health outcomes in clinical (e.g., social anxiety disorder [53],) and non-clinical samples (e.g., [51]) of adolescents, we expect similar pathways liking our variables across all groups (path invariance) and different mean levels between the clinical and the mentally healthy groups (partial mean invariance). Specifically, we expect clinical groups to present higher levels of PI and lower levels of PF compared to the healthy adolescents group.

As a second aim of the current work, we intend to contribute to filling the gap on the efficacy of ACT as a transdiagnostic approach to adolescents' SAD and GAD and amplify the transdiagnostic application of ACT to adolescents presenting these disorders (Study III). To do so, we will adapt, implement, and investigate the efficacy of an ACT Intervention to adolescents presenting SAD or GAD by exploring:

- Changes in primary (i.e., anxiety symptoms) and secondary (i.e., flourishing and PI/PF processes) outcomes following intervention. In view of the scarce but promising research on ACT efficacy in adolescents’ SAD [60, 74, 75], and GAD [60], improvements are expected at post-intervention for the two intervention groups (i.e., SAD and GAD), in comparison with a control group.
- The stability of change over time (i.e., 3 and 6 months after intervention completion). We expect improvements in the two intervention groups to be maintained.
- The equivalence of the efficacy of the same intervention delivered to the two intervention groups (i.e., SAD and GAD). Even though this aim has not yet been addressed, considering ACT conceptual and therapeutic transdiagnostic premises, similar effects on outcome measures are expected for both clinical groups.
- Mechanisms of change following the intervention in both intervention groups. Based on research conducted with adults on PI/PF processes as mechanisms of change following ACT [43], we expect similar effects in both intervention groups, with changes in PI/PF predicting changes in primary (i.e., anxiety symptoms) and secondary (i.e., flourishing) outcome variables. Specifically, we expect increases in PF processes and decreases in PI processes to promote lesser anxiety and greater flourishing. Given the lack of research on the role of each PI/PF process in producing changes in anxiety for adolescents’ GAD and SAD and the ACT assumptions that all processes are intertwined in their contribution to human suffering / flourishing [38], there were no grounds to define specific hypothesis regarding the differential role of each PI/PF process in contributing to decreases in anxiety and increases in flourishing between the two samples of adolescents, and so no specific and process-based hypotheses are put forward.

## Method

This project is funded by the Portuguese Foundation for Science and Technology (Identifier: 2022.13986.BD) and is registered at ClinicalTrials.gov (Identifier: NCT05906849). All procedures were approved by the Ethics Committee of the Faculty of Psychology and Educational Sciences of the University of Coimbra. Eventual protocol amendments will be communicated to this Committee.

### Study design and randomization of participants

**Fig. 1:**
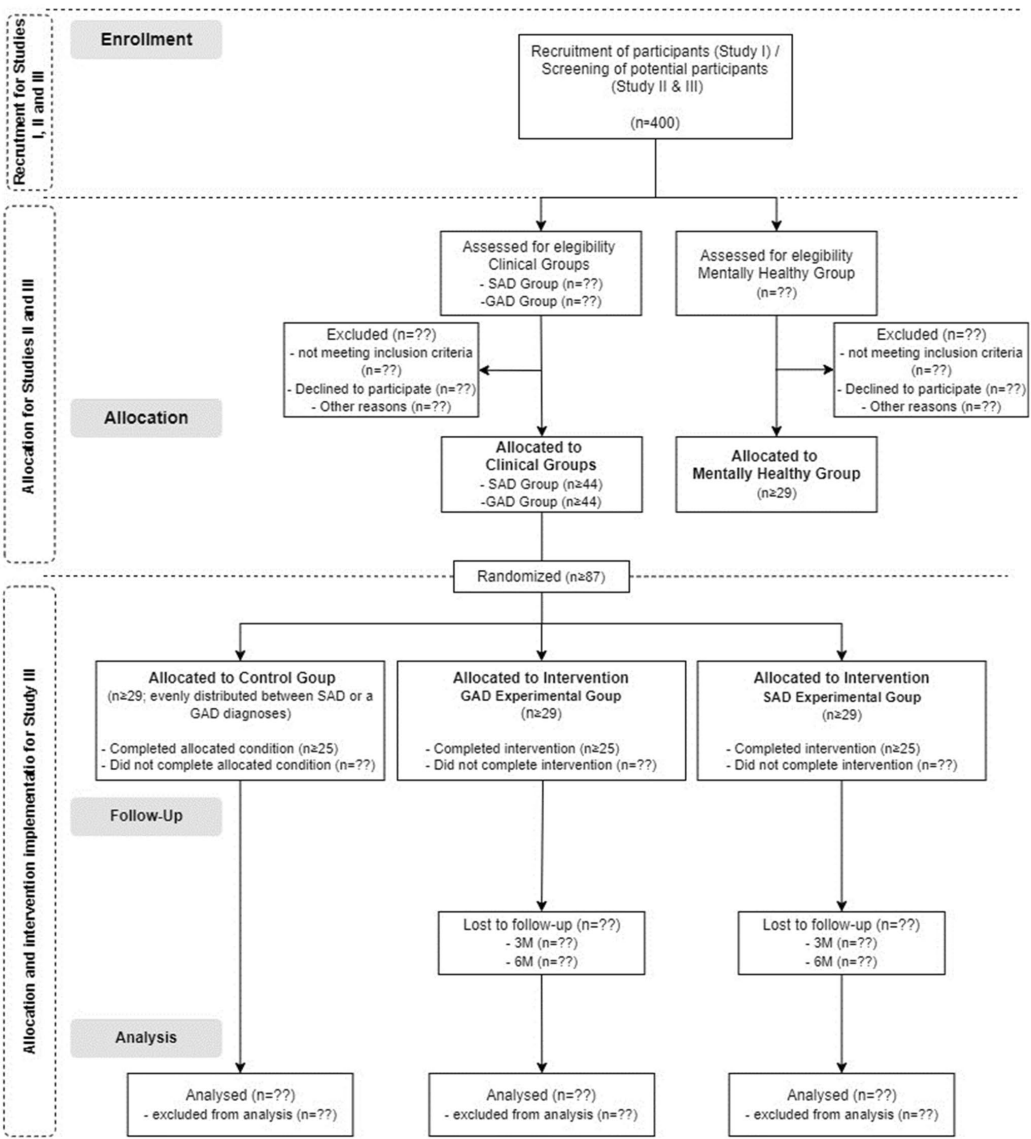
Flowchart of the design and participants allocation for the three sequential studies

The two aims of this research will be accomplished through diverse study designs. To accomplish the first aim, cross-sectional data will be collected from adolescents within a community sample, two clinical groups (with SAD or GAD), and a mentally healthy sample. For the second aim, a Randomized Controlled Trial (RCT) with a parallel design, 3 groups (i.e., Control Group, SAD Intervention Group, and GAD Intervention Group) and 4 assessment moments will be conducted following the CONSORT (Consolidated Standards of Reporting Trials) guidelines [77]. Adolescents from each clinical group from Study II will be randomly allocated (computer-generated random allocation,2:1 allocation ratio) to one of the intervention groups or to the control group. The two clinical intervention groups will receive the same intervention and will be assessed at pre- and post-intervention, and at 3- and 6- month follow-up using a set of self-report measures (see measures section). Adolescents from the control group will be assessed with the same set of self-report measures at 2 different time points (12 weeks interval), mimicking the pre- and post-intervention assessment moments. Given the high stability of both SAD and GAD, we consider ethical obligations to be imperative in not depriving these adolescents of intervention during follow-up assessments. As such, they will be assessed with a clinical structured interview (Mini-Kid; [78]) after the second time point and referred to the school psychology services if the difficulties persist. A flowchart of the study design is presented in Fig. 1.

### Participants recruitment

Participants will be adolescents aged between 14 and 18 years old of both sexes recruited in schools. Informed consent from adolescents and their parents/legal guardians will be required for all potential participants. An initial sample of approximately 400 adolescents from the community will be recruited. All participants will be asked to fill in a set of self-report measures through the *Limesurvey* online platform, and this data will be used for Study I. This set of self-report measures includes the Screen for Child Anxiety Related Disorders (SCARED; [79]) that will be used to screen adolescents to further continue their participation in the study (i.e., Study II and Study III). Specifically, participants scoring higher than one standard deviation above the mean found in a normative sample of adolescents [79] for the Generalized Anxiety or the Social Anxiety subscales of the SCARED (see measures description) will be invited for a clinical structured interview (Mini-Kid; [78],) for the assessment of inclusion/exclusion criteria for the two clinical intervention groups (with SAD or GAD, respectively). Moreover, participants scoring below the mean on the SCARED Reduced Total scale (see measures description) found in the same normative sample [79] will be invited for a clinical structured interview (Mini-Kid [78],) for the assessment of inclusion/exclusion criteria for a group of mentally healthy adolescents. Inclusion criteria for the clinical groups will be presenting a main diagnosis of SAD or GAD, for the SAD and GAD groups, respectively. Inclusion criteria for the mentally healthy adolescents’ sample will be not meeting criteria for a mental health diagnosis. Exclusion criteria for all groups includes presenting cognitive impairment, psychotic symptoms or suicidal ideation, and/or undergoing treatment (e.g., prescribed medication) for a psychiatric condition. Concerning the clinical groups, adolescents presenting both GAD and SAD will be excluded, as its inclusion could confound the study’s results and conclusions concerning the equivalence of ACT for adolescents SAD versus GAD. Adolescents displaying symptoms that warrant attention, but that fall beyond the parameters of this study (e.g., presenting psychotic symptoms, suicidal ideation or both GAD and SAD) will be referred to school psychology services.

### Sample size

Participants recruited in Study II will be randomly assigned to one of the three groups of Study III (cf. Figure. 1). G*Power was used to calculate sample size considering Study III requirements for repeated measures analysis with two groups (considering that the RCT groups will be compared two at a time) and 4 assessment moments. Power analysis for medium effects (f = 0.25) – chosen based on previous ACT studies with adolescents (e.g., [27–30, 60, 80]) –, with a significance level of 0.05 and power of 0.95 requires a sample size of 25 per group. To account for the expected 17% dropout rate reported in a previous meta-analysis of ACT-based interventions [81], the research team intends to collect 4 additional participants per group. Accordingly, Study II will rely on two clinical groups (i.e., SAD and GAD,n ≥ 87) of at least 44 adolescents each, and a mentally healthy group of 29 adolescents (i.e., total n for Study II ≥ 116). For Study III, the clinical participants (i.e., n ≥ 87) will be then assigned to two intervention groups of 29 adolescents each, one for SAD and one for GAD participants, or to a control group of 29 adolescents, evenly distributed between adolescents presenting with SAD or GAD diagnoses.

### Intervention and treatment integrity, feasibility and satisfaction

The same manualized ACT intervention will be delivered to both intervention groups, consisting of 13 individual weekly sessions delivered online through videoconference; this is mandatory for investigating if the same intervention similarly impacts outcomes in participants presenting with diverse diagnostics. The intervention will be developed based on the PF model of ACT [37] and the choice-point model [82], and it will result from the adaptation of the *ACT@TeenSAD* intervention for adolescents with SAD (a 10-session manualized ACT intervention delivered online; Vagos et al., 2021), as well as the integration/modification of sessions’ exercises based on extensive research on ACT, ACT for ADs, and ACT for adolescents, and on the research team’s clinical expertise with adolescents and in delivering ACT based interventions. The ACT intervention for adolescents with ADs will include sessions addressing the functioning of the human mind (e.g., the survival machine, the problem-finding and problem-solving machine), creative hopelessness, the six core processes of PI/PF (e.g., acceptance of difficult thoughts, emotions, and sensations, values clarification), and revision of gains and relapse prevention (cf. Table 1). Each session will have an approximate duration of 1 hour and will be composed of experiential exercises, metaphors, images, and mindfulness practices targeting the specific topic of the session. Participants will be invited to complete between-session tasks as often as they can, in order to practice the strategies addressed in each session (e.g., present moment awareness practices) in real-life contexts. Several aspects will be taken into consideration by the research team members when developing and implementing the intervention to ensure treatment integrity. For example, the research team will seek training in ACT-based approaches, ongoing supervision by experienced psychologists, inclusion of clear and informative descriptions of exercises, as well as therapist lines (e.g., examples of questions to guide adolescents) in the intervention manual, and therapist’s report on treatment adherence after each session.

**Table 1:** Intervention Outline

Session	Contents
1	The human mind and the ACT model: psychoeducation on the nature and functioning of the human mind from an evolutionary perspective, functional assessment, and development of an individualized model to recognize the PI processes maintaining adolescents’ difficulties using the choice-point model
2	Creative hopelessness: reflecting on the costs and workability associate with the control agenda and introducing the possibility of alternative strategies (i.e., brief introduction to the PF processes)
3	Values: exploring the importance of values clarity in one’s life, clarifying the concepts of values (vs goals), identifying adolescents’ personal values and related committed actions (in low anxiety provoking situations)
4	Present moment awareness: reflecting on the role of intentionally contacting the present moment and practicing contact with the present moment in the absence and presence of difficult internal experiences
5	Cognitive defusion: exploring the true nature of thoughts, the impact of hooking and unhooking from them and experimenting different unhooking techniques
6	Acceptance: reflecting on the importance of willingness to contact with difficult emotions and sensations and developing acceptance skills by engaging in multiple exercises
7	Observing-self: introducing the concept of self as a stable and distant observer of the ongoing flow of internal experiences and cultivating this perspective through guided exercises
8–12	ACT informed exposure (committed action): defining stimuli to be exposed to in-session that align with adolescents’ values, identifying obstacles (i.e., PI processes) and helpers (i.e., PF processes related strategies), and conducting exposure exercises
13	Revision of gains and relapse prevention: reflecting on the journey up to this moment, revising the adolescent values and identifying future committed actions, potential obstacles and learned strategies to deal with future difficulties

Sessions will be delivered individually and weekly, with an approximate duration of 1 h each. Metaphors, and/or experiential exercises will be used to address the contents of each session. Between-sessions exercises and practices will also be included

The feasibility of the program will be further assessed through the examination of dropout and adherence rates. Concerning program acceptability and participants’ satisfaction, a questionnaire will be used to assess adolescents’ satisfaction after completing the 13 sessions. The questionnaire comprises 5 items (e.g., “themes addressed in sessions”, “sessions length”) answered on a 5-point Likert scale, assessing the degree to which the participant believes each aspect was adequate, useful and/or relevant (responses ranged from 1 = nothing, to 5 = a lot). This questionnaire also includes three open-ended questions: “what three things did you like the most about the program?” and “what three things did you like the least about the program?” and “if you could change three things about the program, what would it be?”.

### Measures

Participants will be asked to complete a sociodemographic information sheet to assess age and date of birth, biological sex, parent’s professions, area of residence (rural/urban), years of education and grade retentions, existing mental health difficulties (yes/no; if yes, provide a brief description) and current/past psychological support/interventions (yes/no; if yes, describe the motive).

#### Screening and inclusion/exclusion criteria

**Screen for Child Anxiety Related Disorders (SCARED; **[79, 83] The SCARED is a 41-item self-report questionnaire that assesses symptoms of anxiety disorders. Items (e.g., “I worry about other people liking me”) are answered on a 3-point Likert scale (ranging from 0 = ’Almost Never’ to 2 = ’Often’). The SCARED comprises 5 subscales that allow to screen for the following anxiety disorders: generalized anxiety, separation anxiety, somatic/panic, social phobia, and school phobia. The SCARED has shown good internal consistencies, with Cronbach’s alpha values over 0.78 for the total score and subscales [83]. In its Portuguese version [79], the SCARED Total and the SCARED Reduced Total score (i.e., items and scales with poor Cronbach alphas were excluded,consists of 28 items revealed good internal consistencies, with Cronbach’s alpha values of 0.87 and 0.85, respectively. The Generalized Anxiety and the Social Phobia subscales, which will be used in the present study along with the Reduced Total Score, also presented acceptable internal consistencies (i.e., Cronbach’s alpha values of 0.71 and 0.74, respectively; [79]. Evidence for SCARED Portuguese version convergent validity has been found in relation to measures of anxiety and depression symptomatology. Specifically, the SCARED Reduced Total Score and the Generalized Anxiety and Social Phobia subscales presented expected higher correlations with anxiety, compared to the associations with depression [79].

**Mini International Neuropsychiatric Interview for Children and Adolescents (Mini-KID; **[78, 84]). The Mini-KID is a structured diagnostic interview for the assessment of DSM-5 Axis I diagnoses in children and adolescents. Questions answered in a YES/NO format are presented for the evaluation of specific diagnostic criteria for each clinical diagnosis. In the original version of the Mini-KID, interrater reliability was excellent across diagnoses, except for dysthymia [78]. The Portuguese version of the Mini-KID resulted from a careful translation and backtranslation process and has been previously used as a method for the assessment of clinical diagnoses [85].

#### Primary outcomes

**Generalized Anxiety Disorder – 7 (GAD-7; **[86, 87]). The GAD-7 is a 7 item self-report scale initially designed to identify probable cases of Generalized Anxiety Disorder (GAD). Items (e.g., “Feeling nervous, anxious or on edge”) are answered in a 4-point Likert scale (ranging from 0 = ’not at all’ to 3 = ‘nearly every day’) reporting to the two previous weeks, with higher scores representing higher levels of GAD symptoms. In its original version [86], the GAD-7 presented a Cronbach’s alpha of 0.92 and evidence for its construct validity was found in relation to measures of anxiety, depression, and health-related quality of life. In its Portuguese version for children and adolescents [87], the GAD-7 achieved a Cronbach’s alpha value of 0.93, and higher scores on the GAD-7 were associated with lower quality of life and lower satisfaction with school.

**Social Anxiety and Avoidance Scale for Adolescents (SAASA; **[88]**)**. The SAASA consists of 34 items assessing the degree of anxiety and frequency of avoidance in social situations representative of the most frequent social fears during adolescence. Each item (e.g., “Going to a party given by a colleague”) is answered twice, for two subscales – anxiety and avoidance -, on a five-point Likert scale (ranging from 1 = ‘none’ to 5 = ‘very much’, for anxiety; and from 1 = ‘never’ to 5 = ‘almost always’, for avoidance). Each subscale is comprised of six factors: interaction with the opposite sex, assertive interaction, observation by others, interaction in new social situations, performance in social situations, and eating and drinking in public. In its validation study, the SAASA total score and subscales achieved Cronbach’s alpha values over 0.61 [88]. The SAASA has also demonstrated good test–retest reliability, convergent and divergent validities in relation to measures of anxiety, social anxiety and depression, capacity to discriminate adolescents with SAD from adolescents with other anxiety disorders and adolescents without psychopathology [88], and sensitivity to treatment results [89].

#### Secondary outcomes

**Multidimensional Psychological Flexibility Inventory – short form (MPFI—24; **[90, 91**]).** The MPFI-24 is a 24-item self-report scale for the assessment of PI and PF. The scale comprises 12 subscales, representing the processes underlying PF (i.e., Acceptance, Present Moment Awareness, Self as Context, Defusion, Values, Committed Action) and PI (i.e., Experiential Avoidance, Lack of Contact with the Present Moment, Self as Content, Fusion, Lack of Contact with Values, Inaction). Items (e.g., “I opened myself to all of my feelings, the good and the bad”) are answered on a 6-point Likert scale (ranging from 1 = ‘never true’ to 6 = ‘always true’) regarding how true the item was for the respondent in the previous 2 weeks. Each subscale items can be averaged to represent each of the 12 specific dimensions of PF and PI. Likewise, the averages of the 6 flexibility and 6 inflexibility subscales can be averaged to create a composite score representing global flexibility and inflexibility, respectively. Evidence was found favoring the MPFI-24 convergent validity in relation to other measures of PF and PI processes. Also, the subscales and composites have achieved adequate to good internal consistency values [90]. The MPFI-24 Portuguese version for adolescents presented acceptable to good internal consistency values for the subscales and good to excellent internal consistency values for the composites [91].

**Mental Health Continuum – Short Form – for youth (MHC-SF; **[92]**)**. The MHC-SF is a 14 items self-report measure that assesses adolescents’ flourishing based on levels of subjective well-being across 3 domains: emotional, social and psychological well-being. Following the instructions (“Please answer the following questions about how you have been feeling during the past month”), items (e.g., “How often do you felt happy?”) are answered on a 6-points Likert scale (ranging from 0 = ’Never’ to 5 = ’Every day’). In its original version, the MHC-SF domains presented Cronbach’s alphas between 0.78 and 0.84 [93]. In its Portuguese version [92], factors achieved Cronbach’s alpha values ranging from 0.80 to 0.85, and the total score representing flourishing presented a Cronbach’s alpha value of 0.90. Moreover, factors presented expected positive associations with measures of quality of life and life satisfaction, and negative associations with measures of anxiety, depression, and internalizing and externalizing problems [92].

### Data analysis

IBM SPSS Statistics 25 will be used to conduct descriptive analysis. In study I, network analysis will be performed and visualized using R (Version 4.1.3). Network nodes will represent the scores for each PI/PF subscales and total scores for anxiety and flourishing measures. Edges will represent the weight of the correlations between the individual nodes. Centrality metrics (e.g., degree of centrality) will also be computed to investigate how important and influential each node is in the network. In study II, Mplus will be used to investigate the adjustment of a path analysis linking PI/PF processes to anxiety and flourishing, based on overall, comparative and residual based fit-indices. The moderator effect of groups (clinical SAD, clinical GAD and healthy adolescents) in those pathways will be estimated, testing for equality of pathways and means. In Study III, the IBM SPSS Statistics 25 will be used to explore treatment effects using Mixed ANOVAs and considering assessment moments as the within-subject and groups (SAD and GAD receiving intervention and Control Groups) as the between-subject effect; interaction effects will also be considered. Additionally, Mplus will be used to examine mechanisms of change after the intervention by performing two-wave latent change score models. These models allow to explore not only if significant changes emerged across assessment moments, but also to investigate the impact of changes in PI/PF constructs on changes in anxiety or flourishing. All Study III analyses will be carried out according to an intention-to-treat principle as suggested by the CONSORT guidelines [77]. Therefore, all participants randomized will be included in the statistical analyses and analyzed according to the group they were assigned regardless of having received/completed or not the intended intervention. Efforts will be made to obtain outcome data for all participants, even if they do not complete the intervention. If data is missing (e.g., participant refused to provide data, participant was unreachable, etc.) the last observation carried forward method will be employed.

## Discussion

Adolescence is a critical phase of human development where mental health problems, particularly ADs, are highly prevalent (e.g., [3, 4]) and often persist and/or develop into other disorders throughout an individual's life (e.g., [14, 21]). Even if growing evidence points to the importance of understanding the common mechanisms underlying mental health conditions (e.g., [31]), research focusing on adolescents is worryingly limited. Indeed, while PI/PF processes have been largely investigated and ACT has demonstrated to be effective for multiple conditions in adults (e.g., [35, 42]), adolescent populations have been consistently overlooked (e.g., [27–30]). Thus, this project aims to explore the conceptual foundations and therapeutic application of ACT in this pivotal phase of human development. We will delve into the relationships between PI/PF processes, anxiety and flourishing in various adolescents’ samples, and investigate the efficacy of ACT for GAD and SAD in this developmental life stage.

To the best of our knowledge, this is the first project that proposes to comprehensively examine all PI/PF processes in testing ACT assumptions as accurate theoretical conceptualizations of anxiety and flourishing in community adolescents, resorting to network analysis (Study I). Drawing on the theoretical propositions and studies using network approaches to explore associations between PI/PF and mental health outcomes (e.g., [47, 49]), we expect PI processes to display interrelationships and associate positively with anxiety and negatively with flourishing in adolescents from the community. Similarly, PF processes are expected to interconnect with each other and associate negatively with anxiety and positively with flourishing in that community sample. By exploring the strongest and most influential ACT processes in the networks, we will be able to better understand the unique contribution of each PI/PF process within a system, either to flourishing or to anxiety. For example, understanding which process may influence a greater number of other processes, or more strongly impact specific mental health outcomes (e.g., anxiety, flourishing), may help clinicians to increase interventions’ impact. Furthermore, differences in the paths linking PI/PF processes to anxiety and flourishing in clinical (SAD and GAD) and mentally healthy samples of adolescents (Study II) will be explored by testing model invariance. We expect similar pathways linking PI/PF processes to anxiety and flourishing across all groups (i.e., SAD clinical, GAD clinical and mentally healthy groups), with distinct mean levels observed between the clinical and the mentally healthy groups. This would be in line with existing research with adolescents supporting the associations between PI/PF and positive (e.g., [55, 57]) and negative (e.g., [52, 54]) mental health outcomes in clinical (e.g., [53]) and non-clinical samples (e.g., [51]), though not specifically applied to GAD and SAD in adolescence. Moreover, if confirmed, these results will further sustain the role of PI/PF towards adolescents’ mental health, also under a transdiagnostic framework. By investigating specific associations between all PI/PF processes and anxiety as well as flourishing across clinical samples, this research project will provide valuable insights into the necessity for process sensitivity assessment, which may contribute to the development of more tailored and, hopefully, more impactful and idiosyncratic interventions for adolescents.

Additionally, this project will investigate the efficacy of ACT delivered online via videoconferencing to adolescents with SAD and GAD, compared to a control group. The online delivery of ACT is a relevant strength of this research project. The efficacy of full synchronous online ACT interventions delivered to adolescent SAD and GAD have not yet been investigated. However, this method of delivering interventions may be particularly relevant to adolescents as it is more accessible and it may help to circumvent obstacles to treatment seeking such as autonomy (i.e., being dependent on parents/adults to drive them to clinics), time and money spent on commuting and fear of stigmatization when seeking in-person psychological support [94]. Moreover, adolescents spend more time online than any other age group, and so online interventions have the potential of being a well-received method, thus enhancing the delivery of mental health care within this adolescence [95].

By using a robust RCT design to investigate the efficacy of ACT, this project will address the scarcity of methodologically robust designs examining that topic specifically in relation to mental health conditions in adolescents (e.g., [27–30, 65]). We will also compare the efficacy of the same ACT intervention between GAD and SAD clinical groups, expecting similar effects on outcome measures. Moreover, we will investigate the maintenance of intervention gains over time, which will offer a better understanding of the utility of ACT during a period of significant and rapid physical, psychological, and social changes. If the intervention is found to be efficacious, this project will support the future delivery of ACT interventions to adolescent ADs as a reliable transdiagnostic alternative, and as an added option for those who do not achieve desired results from standard treatments. This may offer clinicians the opportunity to choose interventions based on adolescents needs, while adhering to evidence-based treatments and benefiting from the practical and economical implications of transdiagnostic approaches in clinical practice.

Finally, to the best of the authors' knowledge, this project will be the first to explore mechanisms of change following an ACT intervention for adolescents with SAD and GAD. We will consider all PI/PF processes, providing a more comprehensive examination of potential change mechanisms. This finding will further support the theoretical consistency of the ACT model and shed light on the processes responsible for change, thereby expanding the reach and benefits of ACT interventions. Based on previous research on the relevance of the ACT processes [43, 44] and on the transdiagnostic model underlying ACT (e.g., [37, 38]), we expect changes to those processes to predict changes in flourishing and anxiety symptoms similarly in both clinical intervention groups. From an ACT perspective ([39, 38]), adaptative behavioral change results from reducing inflexible patterns in which private events heavily influence action taking, distancing individuals from a valued living (PI) through cultivating the ability to fully contact the present moment as it is and persist in and/or change behavior in ways that bring meaning and vitality to one’s life (PF). Therefore, we expect decreases in PI processes and increases in PF processes to account for higher levels of flourishing and lower levels of anxiety following the intervention. This expectation aligns with the clinical features of many anxiety disorders, such as GAD and SAD, where avoidance of anxiety-triggering situations and rumination about future events are key maintenance factors encompassed within PI processes such as inaction, impulsivity, or avoidant persistence, and dominance of the conceptualized past/feared future. About specific PI/PF process, equal importance is attributed to them within the ACT framework, as the inflexible/flexible patterns of responding to internal experiences and contextual cues and engaging in unworkable/workable behaviors result from the interdependent interactions among them that sustain overall PI/PF ([38]). For instance, helping adolescents see their thoughts as mental events rather than literal representations of reality (cognitive defusion), may help foster present moment awareness which, in turn, creates space for them to choose to avoid less anxiety triggering situations and take more contextual useful and values guided actions [96]. At the empirical level, it remains unclear the extent to which each individual PF/PI process may be more or less important to promote change, particularly while accounting for all model processes (see [43] for a review). Thus, we did not make specific predictions about which PI/PF processes would most influence change in anxiety or flourishing, as all processes are expected to contribute to change.

This study presents some foreseen limitations that are, nevertheless, necessary for its viability. Firstly, it focuses solely on SAD and GAD, disregarding other ADs that also present significant prevalence rates and impairments during adolescence. Additionally, the control group will not receive intervention within this project. To ensure these adolescents receive appropriate care, they will be assessed after completing the control condition and referred to school psychology services if difficulties persist. They will also be explicitly informed about the possibility of discontinuing their participation in the study to seek treatment, without any consequences. It is also important to note that the control group may not be entirely comparable to either of the intervention groups, which could lead to inherent disparities and the introduction of potential confounding variables. Based on ACT transdiagnostic premises, we believe that these differences will not significantly influence our findings. Nevertheless, the authors have devised a contingency plan to address this potential limitation, if necessary (i.e., gathering two independent control groups: one consisting solely of adolescents with SAD and another comprising only adolescents with GAD). We also acknowledge that by only using self-report measures, data collection may be susceptible to the effects of social desirability, and only a limited comprehension of the psychological constructs assessed is provided. Moreover, we anticipate dropouts to be a significant challenge in this study. To prevent its impact, the research team plans to recruit more participants than the minimum needed for each condition. This approach will allow us to account for potential attrition and ensure that sample size remains adequate throughout the study, maintaining statistical power. Efforts will be made to engage and motivate participants throughout the duration of the study to maximize retention rates (e.g., by sending thank you messages after each assessment moment). Lastly, we acknowledge that by not considering a group with comorbid GAD and SAD, we cannot fully elucidate the applicability of transdiagnostic principles to adolescents. Future research should consider comorbidity and/or cascading effects of mental health difficulties as relevant outcomes to better address the complex issues related to ACT as applied to adolescent mental health.

Albeit these anticipated limitations, we believe that this project holds great relevance in advancing knowledge of transdiagnostic processes underlying psychological functioning in adolescents. It also has the potential to inform and enhance the efficacy of interventions for this population. ACT shows promise as a therapy for adolescents with ADs (e.g., [60]), as its underlying transdiagnostic premises may help mitigate the impact of present and future mental health conditions. By exploring PI/PF processes in both clinical and non-clinical adolescent populations and testing the efficacy of ACT along with mechanisms of change, we will gain a better understanding of how (i.e., through which specific PI/PF processes) and for whom (i.e., GAD and SAD) ACT can contribute to adolescents’ mental health. This knowledge will ultimately contribute to the development of more effective and tailored interventions for this population, with the potential to improve their well-being and long-term mental health outcomes.

## Supplementary Information

Below is the link to the electronic supplementary material.

Supplementary file1 (DOCX 19 KB)

## Data Availability

No datasets were generated or analysed during the current study.
