# Supplementary material for: Acceptance and commitment therapy as a transdiagnostic approach to adolescents with different anxiety disorders: study protocol
Source: Eur Child Adolesc Psychiatry. 2024 Nov 14;34(6):1931–44. doi: 10.1007/s00787-024-02608-2 (PMC12198063; doi:10.1007/s00787-024-02608-2)
Supplement: Supplementary file 1 — Supplementary file1 (DOCX 19 KB) [file 787_2024_2608_MOESM1_ESM.docx]

| **Table 2. Psychological flexibility and inflexibility processes descriptions** | | | |
| --- | --- | --- | --- |
| **Psychological Inflexibility processes** | | **Psychological Flexibility processes** | |
| **Cognitive Fusion** | Thoughts are perceived as literal representations of reality and dominate awareness, thus rigidly guiding behavior away from a meaningful life | **Cognitive Defusion** | Noticing and observing thoughts as passing mental events rather than rigidly accepting them as objective truths, thereby reducing their influence on behavior |
| **Experiential Avoidance** | Attempts to control, avoid, or suppress the form, frequency, or intensity of unpleasant internal experiences (e.g., thoughts, emotions) when doing so is unhelpful to pursue value-guided actions | **Acceptance** | Willingness to allow difficult internal experiences (e.g., thoughts, emotions) to arise without attempting to avoid, control, or suppress them |
| **Attachment to the Conceptualized Self** | Becoming overly identified with one's self-descriptions, evaluations, and judgments, perceiving them as a definitive representation of one's true identity, which inhibits behavior change | **Self as Context** | Observing private events from a perspective that is distinct and separate from one's core sense of self, allowing for behavioral adaptability and change |
| **Dominance of the Conceptualized Past/Feared Future** | Tendency to get stuck in the past/future leading to actions guided mostly by cognitive products of the mind than by the present-moment reality | **Contact with Present Moment** | Consciously directing attention to private and external experiences as they occur in the present moment in a nonjudgmental way |
| **Lack of Values Clarity** | Being disconnected from one's important life directions, resulting in a lack of clear guidance for engaging in meaningful and effective actions that align with personal values | **Values** | Clear understanding of and connection with personally meaningful life directions that guide decision-making and action |
| **Inaction, Impulsivity or Avoidant Persistence** | Behavior repertoire characterized by impulsive, reactive, or automatic actions often driven by the avoidance of uncomfortable experiences and the fusion with unhelpful thoughts diverting individuals from pursuing a meaningful life | **Committed Action** | Consistently taking actions that align with one's deeply held values, even when faced with discomfort, and adjusting as needed to remain true to those values |
| *Note.* For further elaboration on each process see Hayes et al. (1999, 2006) | | | |

**Supplementary material**
